# Modified stepping behaviour during outdoor winter walking increases resistance to forward losses of stability

**DOI:** 10.1038/s41598-023-34831-3

**Published:** 2023-05-24

**Authors:** Aaron N. Best, Amy R. Wu

**Affiliations:** Mechanical and Materials Engineering, Ingenuity Labs Research Institute, Queen’s University, Kingston, K7L 2N9 Canada

**Keywords:** Mechanical engineering, Biomedical engineering

## Abstract

Healthy humans are proficient at maintaining stability when faced with diverse walking conditions, however, the control strategies that lead to this proficiency are unclear. Previous laboratory-based research has predominantly concluded that corrective stepping is the main strategy, but whether this finding holds when facing everyday obstacles outside of the laboratory is uncertain. We investigated changes in gait stability behaviour when walking outdoors in the summer and winter, hypothesizing that as ground conditions worsened in the winter, the stepping strategy would be hindered. Stability would then be maintained through compensatory strategies such as with ankle torques and trunk rotation. Data was collected in the summer and winter using inertial measurement units to collect kinematics and instrumented insoles to collect vertical ground reaction forces. Using the goodness of fit for a multivariate regression between the centre of mass state and foot placement we found that, counter to our hypothesis, stepping was not hindered by winter conditions. Instead, the stepping strategy was modified to increase the anterior-posterior margin of stability, increasing the resistance to a forward loss of stability. With stepping being unhindered, we did not observe any additional compensation from the ankle or trunk strategies.

## Introduction

Healthy humans are able to navigate complex environments in day-to-day life while maintaining stability and avoiding falls. However, as we age the ability to maintain stability worsens, and falls become more prevalent. These falls seem to occur more commonly when weather conditions are worsened. Morency et al.^1^ tracked the location of falls that required ambulance assistance. They found that 47% of the 960 outdoor falls reported took place during times when ice formation was increased by a rapid decrease in temperature or freezing rain. The occurrence of these outdoor falls can be further exacerbated by age. A study from Bergland et al.^2^ of 307 women aged 75 and older found that 57.5% of the 308 reported falls over one year occurred outdoors. Despite the knowledge that outdoor falls occur at a higher rate due to poor weather conditions, it is still unknown how healthy humans adapt their gait to maintain stability in these conditions which limits our understanding of fall prevention.

Studies of indoor walking have shown that stability is maintained through the control between the centre of mass (CoM) and base of support (BoS)^3^. This stability can be achieved with three main strategies^4^: the stepping strategy, the ankle strategy, and the trunk strategy. The stepping strategy is when an individual takes a corrective step, or alters the intended location of a step during walking in order to change the BoS and maintain stability^5^. The ankle strategy is when an individual generates torques about the ankle joint to move the centre of pressure (CoP) under the foot to alter the acceleration of the CoM^4,6^. The final strategy is the trunk strategy when an individual generates torques at the hips, moving the large mass of the trunk, which in turn affects the placement of the body CoM^4,6^. During walking, healthy humans can use some combination of all these strategies to maintain stability and overcome perturbations.

Previous research conducted in indoor settings has aimed to identify the contributions of each of the different strategies. They have mainly concluded that the stepping strategy is the primary means by which stability is maintained^5^ and that the foot placement is highly correlated with the previous state of the CoM^7^. However, other studies suggest the ankle and trunk strategy also have non-negligible contributions to stability. Hof et al.^8^ found that, in the frontal plane, step placement was able to compensate for larger perturbations than the ankle strategy (approximately 10 times greater), but the reaction time of the ankle strategy was much faster (approximately 100 ms faster). Similarly, Vlutters et al. found that in the ML direction foot placement was the main strategy used to maintain stability^9^. However, in the AP direction stepping was not solely responsible for maintaining stability and likely the ankle strategy was used to provide additional compensation. In a follow-up study, Vlutters et al. restricted the ankle strategy by using pin-shoes that reduced the foot contact area^10^, resulting in adjustments to the stepping strategy, but no additional compensation from the trunk strategy was found in the AP direction. Few studies of walking have provided insights into the contributions of the trunk strategy to ML stability during walking. In a prior study of very slow walking^11^, we found a larger contribution of the trunk and ankle strategies in the ML direction to compensate for the increased amplitude of the ML CoM at very slow speeds (less than 0.6 m/s). A recent study from van den Bogaart et al.^12^ found an increased contribution of the trunk strategy when both the stepping and ankle strategies were restricted in the ML direction. Although these experiments have provided meaningful insights into the contributions and compensation of the different strategies, they all took place indoors where stepping is typically unrestricted and the ground is able to provide adequate support. These favourable conditions might not occur in the walking environments faced outdoors.

Previous studies have recreated aspects of outdoor conditions in indoor labs, including uneven terrain^13–15^ and reduced traction^16^. The results of these studies are not uniform, however, and the various simulated environments tested also preclude broad conclusions about walking over natural terrain. Voloshina et al.^13^ studied walking on uneven terrain using rectangular wooden blocks of different height covered in foam. They found that the uneven surface caused an increase in step width variability and stride length variability, a decrease in stride length, and no significant change in step width. Gates et al.^15^ compared the frontal plane dynamics of healthy individual and individuals with transtibial amputations while walking on loose rocks. In healthy individuals, they found an increase in both the mean value and variability of step width but no change in the mean ML margin of stability (MoS)^3^. The ML MoS variability, however, was increased by the loose rock surface. Similarly, Curtze et al.^14^ found that the mean step width increased and mean ML MoS remained the same when walking on an irregular surface. Bone et al.^16^ investigated the effect of walking on a known slippery surface on balance control. When the subjects expected to slip, they altered their AP MoS by reducing their stride length and increasing the distance from the extrapolated CoM (XCoM)^3^ to the posterior edge of the BoS, increasing the resistance to a backwards loss of stability. Although these studies may be able to emulate some of the features of the conditions outdoors, they do not necessarily encompass all of the changes that may be present. For example, ground conditions during the winter may be both uneven and slippery due to ice and snow.

The few research studies on walking outdoors^17–19^ have utilized inertial measurement unit (IMU) based motion capture for remote data collection outside fixed laboratory environments. Matthis et al.^17^ investigated how humans use their gaze to plan their next step while walking on difficult terrains. Schmitt et al.^19^ compared walking indoors, outdoors, and on a treadmill and found that when walking outdoors, subjects walked at a faster pace with longer and more variable stride lengths. Kowalsky et al.^18^ investigated how the terrain affected different gait parameters and energy expenditure during walking in the real world. They found that spatiotemporal measures and energy expenditure were affected by terrain and that the changes in spatiotemporal measures could predict energy expenditure. Although these previous studies have provided some insights into the broad gait changes induced by outdoor walking, they do not specifically address gait stability and the gait adaptations and balance strategies humans might employ to navigate real-world conditions.

Here we investigated how healthy humans adapt their gait when walking in everyday outdoor conditions. Specifically, we compared differences in gait stability behaviour between summer and winter weather conditions and expected to find changes in both stability and spatiotemporal measures. We hypothesized that the winter conditions will reduce the available locations for foot placement, affecting ML and AP stability, spatiotemporal measures, and possibly require additional compensation from the ankle and trunk strategies. Consistent with prior literature on simulated terrains, we expected that the mean ML MoS would remain constant while the AP MoS would change to create a greater resistance to a backwards loss of stability in the winter conditions. In both the ML and AP directions, we expected an increase in the MoS variability in the winter conditions. Due to the changes expected from our primary hypotheses on MoS, we also expect changes in spatiotemporal measures. Specifically, we predicted that when faced with uneven and slippery conditions in the winter, subjects will elect to decrease stride length while increasing step width, stride length variability and step width variability. The increase in step width will coincide with an increase in the lateral excursion of the CoM, causing the constant ML MoS we previously hypothesized.

## Methods

We conducted experiments on healthy subjects walking outdoors during the summer and winter months in Kingston, Ontario, Canada. During the experiments, body kinematics were collected using a full body IMU-based motion capture system, and vertical ground reaction forces were collected using instrumented insoles. From these experiments, we obtained normalized stride-by-stride data of subject behaviour while walking in the different environmental conditions. Using this normalized data, we analyzed the changes in body kinematics, spatiotemporal measures, ground reaction forces (GRF), stability measures, and the contributions of the different stability strategies brought about by the changes in walking conditions.

### Experiment

Seventeen healthy, young adult subjects ($N=17$, 7 female, 10 male, weight 74.0 ± 10.3 kg, height 1.74 ± 0.06 m, age 20–33 years) participated in the study with 14 subjects taking part in both the summer and winter experiments and the remaining 3 subjects only participating in one of the experiments. With these subjects, a total of 31 experiments were conducted with 15 data collections in the summer months and 16 data collections in the winter months. All subjects provided informed consent, and the experiments were approved and conducted in accordance with ethics review board of Queen’s University. Data from one subject in the winter could not be used due to the poor quality of the IMU sensor calibration. Due to an equipment malfunction, insole data from the winter trials of six subjects were removed, but all their other data remained for analysis.

**Figure 1:**
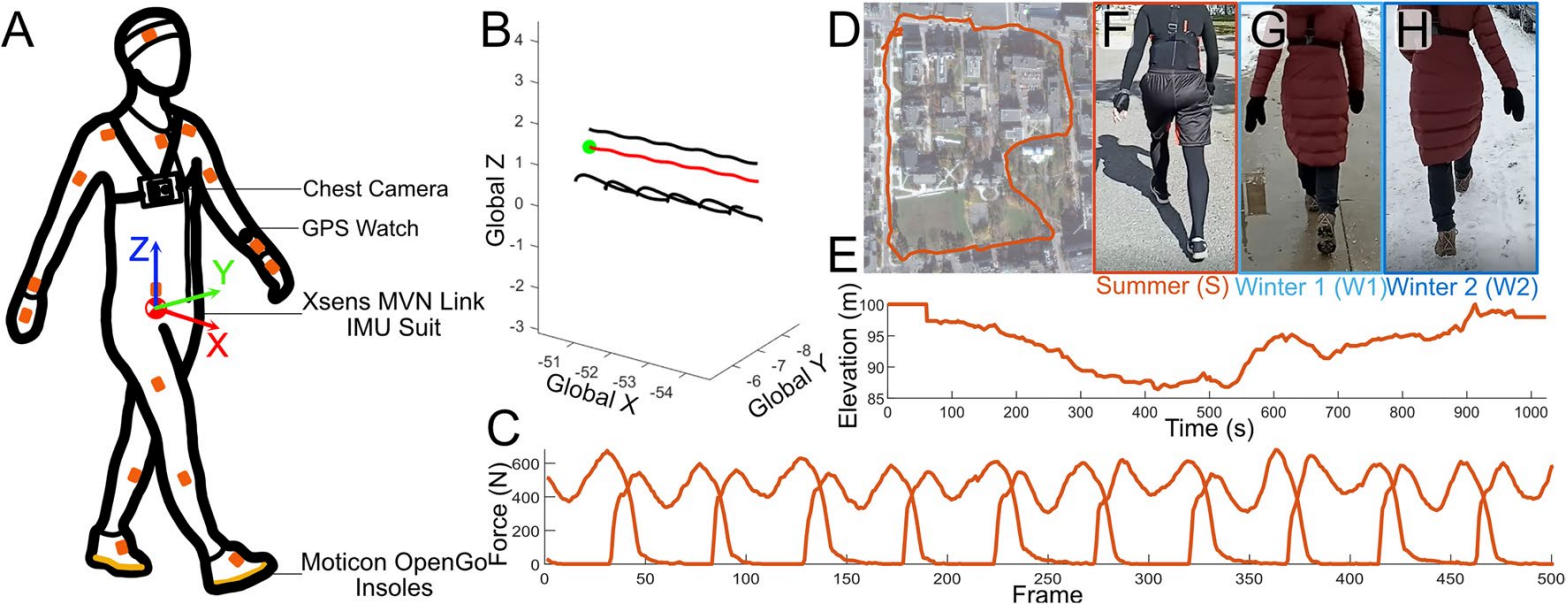
Instrumentation and walking terrain of the subjects for the experiments. (**A**) Schematic representation of the instrumentation of the subjects, including the Xsens MVN Link IMU suit, Moticon OpenGo instrumented insoles, GPS watch and chest mounted camera. Coordinate system shown is an example of the stride coordinate system created for each individual stride. (**B**) Exemplar trajectories of the CoM (red), C7, and heels, provided by the IMU suit during a summer experiment. (**C**) Example force measurements from instrumented insoles during a summer experiment. (**D**) Top down view of the path provided by the GPS watch during a summer experiment. The same path was used for the winter experiment. (**E**) Example elevation of the subject as a function of time during the experiment while walking during a summer experiment. (**F**–**H**) Examples of the terrain that was categorized into the different conditions with images from the videos recorded by the experimenter following the subject. Condition S (**F**) was for the entirety of the summer experiments. Condition W1 (**G**) was for the strides in the winter experiments in which the path was sufficiently plowed and salted such that it was predominantly free of ice and snow. Condition W2 (**H**) was for the strides in the winter experiments in which the ground was predominantly covered in ice, snow, and slush.

The experiments took place in Kingston, Ontario, Canada, where the weather conditions are dissimilar between the summer and winter months, with an average temperature difference of $27.4\,^{\circ }$C^20^ between the two data collections. The summer data collection took place from August 16, 2021 to October 19, 2021 where the temperature during the data collections ranged from $26\,^{\circ }$C to $10\,^{\circ }$C (mean: $17.8 ^{\circ } \pm 4.8 \,^{\circ }$C)^20^. In the summer, the walking surface is consistently dry concrete during data collection and thus has fewer obstacles on the ground (Fig. 1F). The winter data collection took place from January 19, 2022 to March 03, 2022, where the temperatures during the data collections ranged from $2\,^{\circ }$C to $-24\,^{\circ }$C (mean: $-9.6^{\circ } \pm 7.4\,^{\circ }$C)^20^. In the winter months the ground conditions are more inconsistent compared to the summer. There are areas that have been shovelled and treated with salt to remove snow and ice, producing a ground condition that is predominantly wet concrete (Fig. 1G). There are also other areas where the snow and ice have not been sufficiently cleared, creating a ground condition that is predominantly covered in ice and snow which may limit the available stepping locations (Fig. 1H). There was no control or alteration of the conditions present during the data collection. The experiments took place along the same path regardless of the conditions that were present.

**Figure 2:**
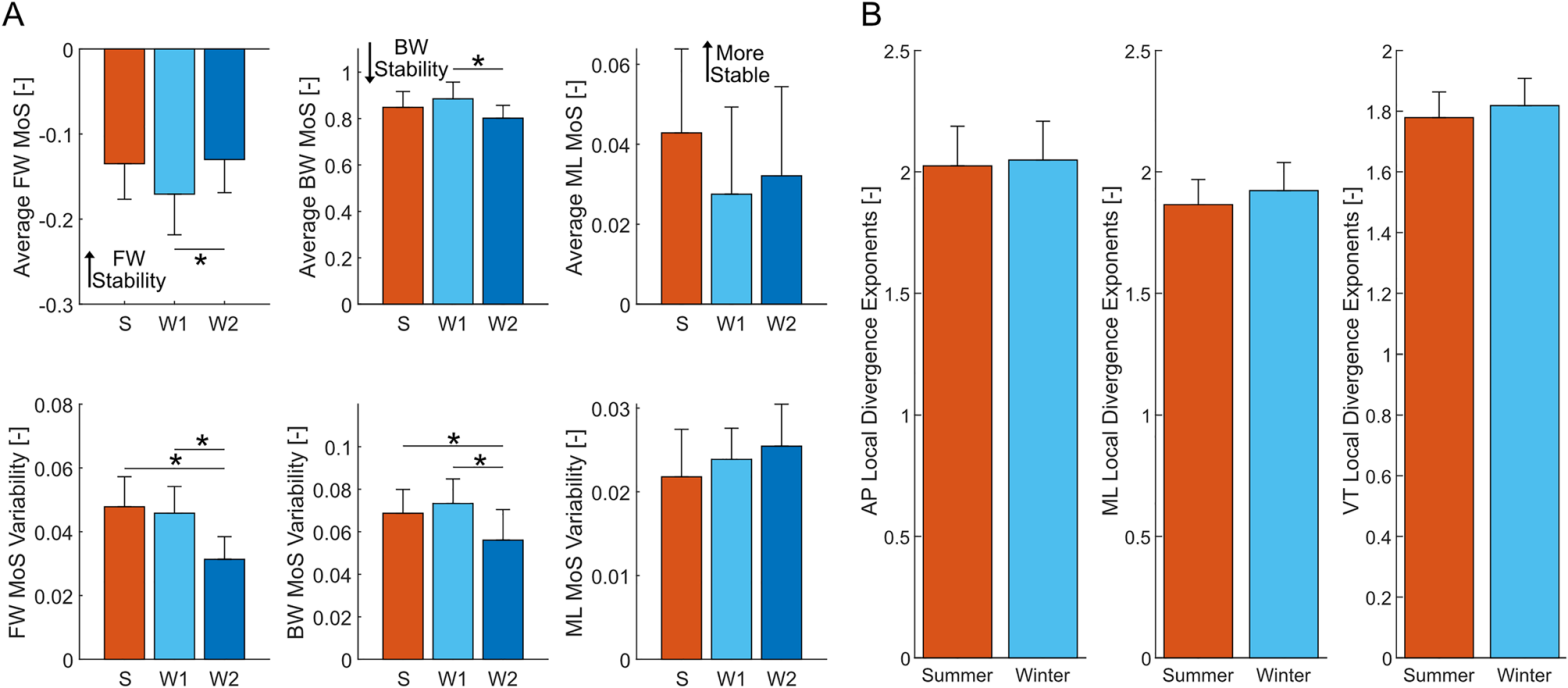
Margin of stability (MoS) and local divergence exponent (LDE) across the different experiments and conditions. (**A**) Mean and standard deviation of the mean MoS and MoS variability in the AP and ML direction in condition S ($N = 15$), condition W1 ($N = 15$), and condition W2 ($N = 14$). The AP direction includes forward (FW) MoS and backward (BW) MoS. (**B**) Mean and standard deviation of the LDE in the anterior-posterior (AP), mediolateral (ML), and vertical (VT) directions for the summer and winter experiments. Bars are the average values across all subjects, and the error bars denote 1 s.d. The asterisks denote statistically significant differences between conditions ($p<0.05$).

**Figure 3:**
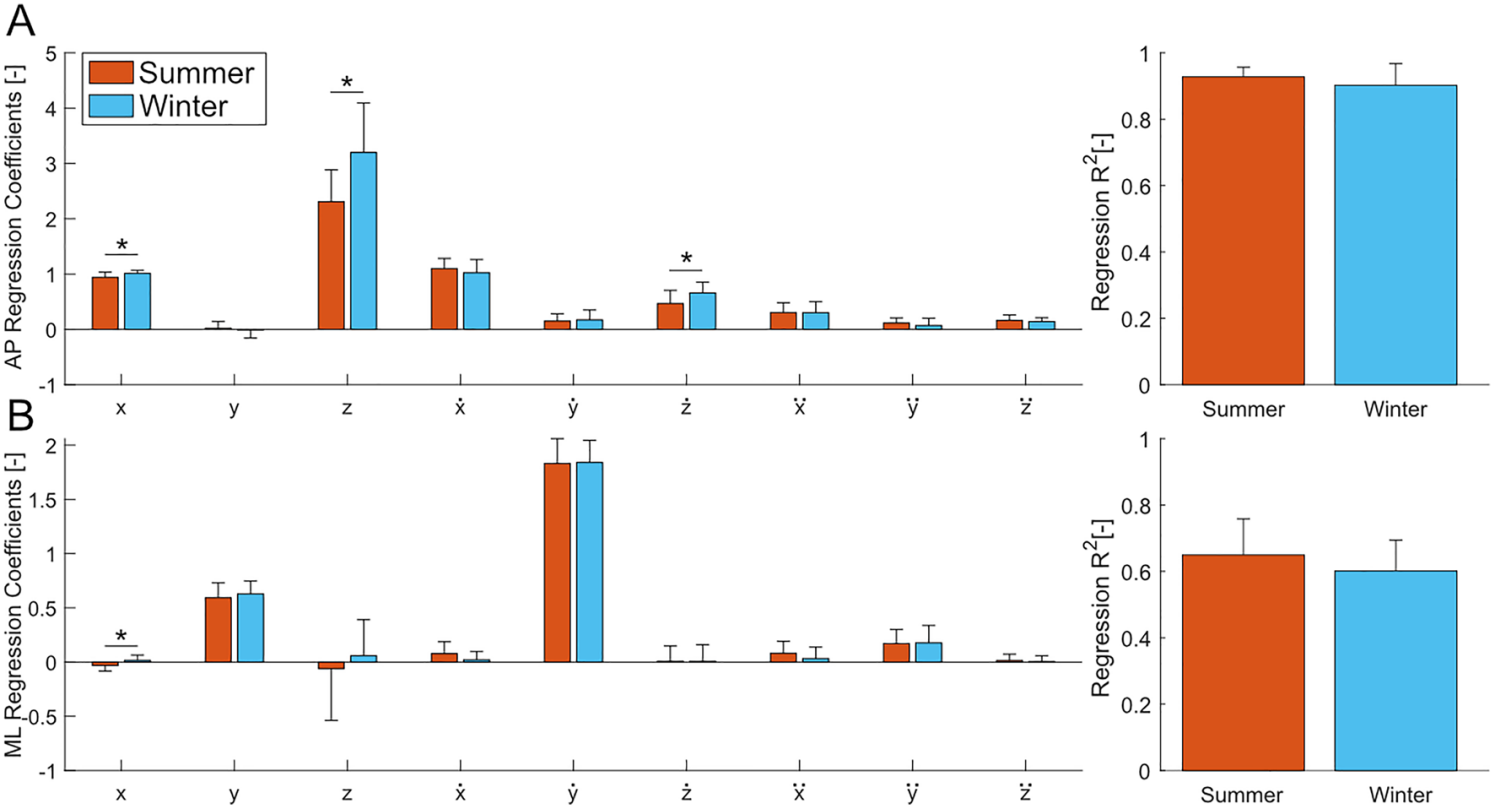
Coefficient and $R^2$ values for the stepping regression in the AP and ML direction for the summer and winter experiments. (**A**) Mean and standard deviation of the regression coefficients and the $R^2$ values for the AP regression. (**B**) Mean and standard deviation of the regression coefficients and the $R^2$ values for the ML regression. Bars are the average values across all subjects ($N = 15$), and the error bars denote 1 s.d. The asterisks denote statistically significant differences between conditions ($p<0.05$).

**Figure 4:**
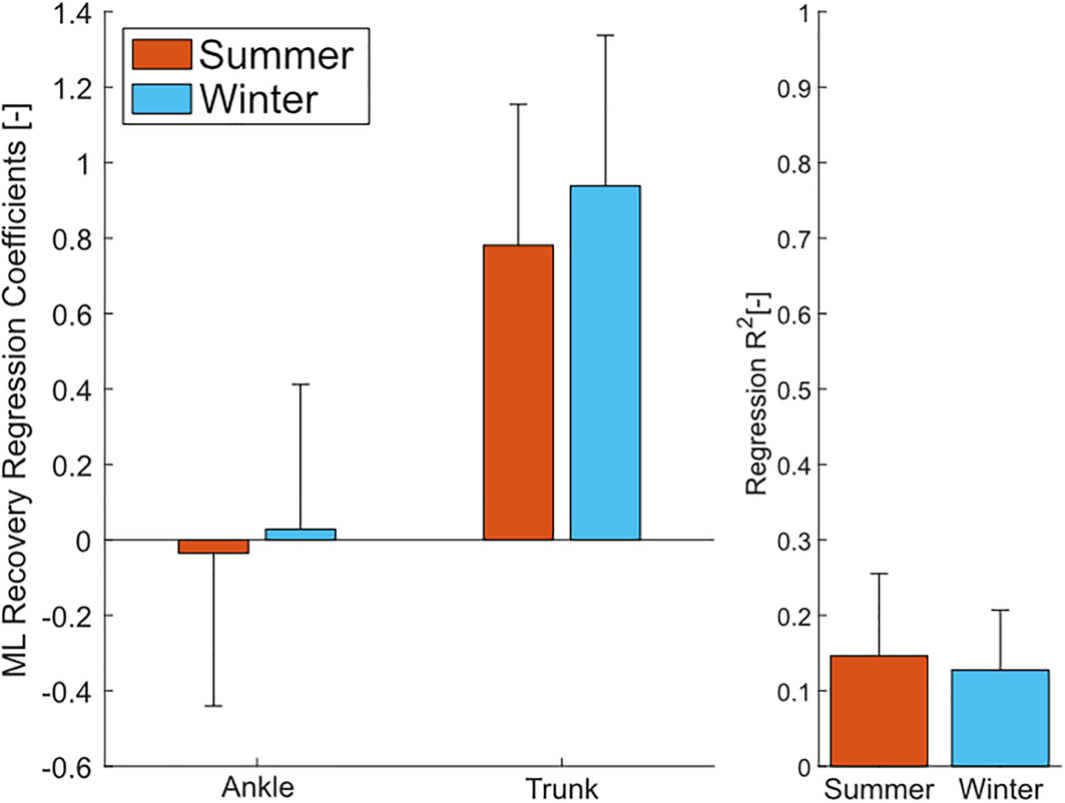
ML ankle and trunk strategy regression coefficients and $R^2$ values for the summer ($N = 15$) and winter ($N = 9$) experiments. Bars are the average values across all subjects, and the error bars denote 1 s.d.

During the experiment, subjects wore a full-body IMU-based motion capture suit (MVN Link, Xsens, Enschede, Netherlands) to capture kinematic data (Figure 1A,B). This suit consisted of 17 IMUs that were placed on the lower and upper body on segments required by the Xsens software^21^. Additionally, subjects wore instrumented insoles (OpenGo, Moticon, Munich, Germany) to record the CoP and vertical GRF (Fig. 1A,C). The IMU data was sampled at 240 Hz while the insole data was sampled at 100 Hz. Subjects also wore a GPS tracking watch (Instinct, Garmin, Olathe, USA) to record the coordinates of the walking path (Fig. 1D,E) and a chest mounted camera to record any ground level obstructions (Fig. 1A). For the summer data collections, subjects were instructed to bring running shoes, and in the winter, subjects were instructed to bring shoes that they would typically wear during winter months with laces if possible for ease of IMU attachment. In the winter months, subjects wore winter jackets and additional clothing as needed over the IMU suit to stay warm.

**Table 1:** Mean and standard deviation of the stability and regression measures for condition Summer ($N = 15$) and condition Winter ($N=15$), or condition W1 ($N = 15$) and condition W2 ($N = 14$).

Stability and regression measures	Summer (S)	Winter 1 (W1)	Winter 2 (W2)	*P* ($\eta ^2$)
FW MoS mean	− 0.1349 ± 0.0418	− 0.1706 ± 0.0479	− 0.13 ± 0.0389$^{W1}$	0.0280* (0.1600)
FW MoS variability	0.0478 ± 0.0094	0.0458 ± 0.0084	0.0314 ± 0.0071$^{S,W1}$	5.5834e−6* (0.4457)
BW MoS mean	0.8484 ± 0.0683	0.8852 ± 0.0722	0.8018 ± 0.0552$^{W1}$	0.0060* (0.2209)
BW MoS variability	0.0687 ± 0.0112	0.0733 ± 0.0116	0.0561 ± 0.0144$^{S,W1}$	0.0018* (0.2662)
ML MoS mean	0.0428 ± 0.021	0.0275 ± 0.0218	0.0321 ± 0.0223	0.1527
ML MoS variability	0.0218 ± 0.0057	0.0239 ± 0.0037	0.0255 ± 0.005	0.1382

	Summer	Winter		
AP LDE	2.0249 ± 0.1633	2.0488 ± 0.1601		0.6881
ML LDE	1.8651 ± 0.1036	1.9229 ± 0.1157		0.1600
VT LDE	1.7788 ± 0.0847	1.8184 ± 0.0897		0.2251
AP regression $R^2$	0.9277 ± 0.029	0.9025 ± 0.0648		0.1816
ML regression $R^2$	0.6495 ± 0.1089	0.6017 ± 0.0921		0.2042
ML recovery regression $R^2$	0.1463 ± 0.1089	0.1275 ± 0.0796		0.6570

The value of *P* represents the p-value from one-way ANOVA (S, W1, W2) or t-test (Summer, Winter) as appropriate with statistical significance ($p<0.05$) indicated by asterisks. Statistically significant results are accompanied with their respective effect size ($\eta ^2$). The superscripts ($^{S}$ or $^{W1}$) indicate the condition from which there were significant differences found from post-hoc t-tests.

**Table 2:** Mean and standard deviation of spatiotemporal measures for condition S ($N = 15$), condition W1 ($N = 15$), and condition W2 ($N = 14$).

Spatiotemporal measures	Summer (S)	Winter 1 (W1)	Winter 2 (W2)	*P* ($\eta ^2$)
Stride time	3.5145 ± 0.2254	3.4045 ± 0.2352	3.4186 ± 0.2329	0.3766
Stride time variability	0.0802 ± 0.0169	0.0809 ± 0.0219	0.0861 ± 0.0308	0.7688
Stance time	2.1198 ± 0.1546	2.0161 ± 0.1665	2.0988 ± 0.1621	0.1899
Stance time variability	0.0987 ± 0.0214	0.0907 ± 0.0250	0.0693 ± 0.024$^{S,W1}$	0.005* (0.2269)
Speed	0.5128 ± 0.0410	0.5283 ± 0.0346	0.5086 ± 0.0355	0.3279
Speed variability	0.0214 ± 0.0041	0.0228 ± 0.0050	0.0219 ± 0.0071	0.7794
Stride length	1.7711 ± 0.0945	1.7677 ± 0.0787	1.7106 ± 0.0823	0.1166
Stride length variability	0.0601 ± 0.010	0.0673 ± 0.0252	0.0702 ± 0.0254	0.4111
Step width	0.0988 ± 0.0457	0.0832 ± 0.0514	0.0932 ± 0.0533	0.6911
Step width variability	0.0346 ± 0.0037	0.0364 ± 0.0066	0.0373 ± 0.0074	0.4604

The value of *P* represents the p-value from one-way ANOVA (S, W1, W2) with statistical significance ($p<0.05$) indicated by asterisks. Statistically significant results are accompanied with their respective effect size ($\eta ^2$). The superscripts ($^{S}$ or $^{W1}$) indicate the condition from which there were significant differences found from post-hoc t-tests.

Subjects were asked to walk normally, which included avoiding any obstacles or unfavourable terrain that they deemed appropriate to avoid. In the event of a loss of balance without falling, subjects were asked to briefly raise their hand to indicate that a slip had occurred. Prior to beginning the experiment, subjects were shown the intended route (Fig. 1D). Once the experiment had begun, the subjects were then followed from behind by the experimenter who was filming the trial to track ground conditions. Periodically during the experiment, the experimenter would remind the subject of the upcoming direction to ensure that the subject did not stray from the intended route. At the beginning and end of the experiment, the subjects were asked to jump in place three times to synchronize the insoles with the IMU suit. The recommended calibrations^21^ for the IMU suit were conducted multiple times before and after the experiment. The repetition of the calibration was to ensure that there was at least one acceptable calibration to use in the processing of the outdoor walking data.

### Analysis

The IMU data was processed using the Xsens MVN software to obtain full body kinematics^22,23^, which was then downsampled to 120 Hz. Similarly, the data from the insoles was processed by the Moticon SCIENCE software to obtain the ground reaction force and CoP. No loss of balance events were self-reported by the subjects, and therefore, no additional analysis was performed on these events.

The kinematic data from the IMU suit was divided into strides (820 strides per experiment on average) and then subsequently rotated and translated such that the motion aligned with the anatomical directions. This was done be creating an individual coordinate system for each stride in which the x, y, and z axes aligned with the AP, ML, and VT directions, respectively (Fig. 1A), referred to as the stride coordinate system. The heel strike and toe off gait events used to separate this data were found using the contact detection provided by the Xsens MVN software. The locations of key anatomical features were transformed into the stride coordinate system and normalized to percent gait delineated by right heelstrike. The key anatomical features extracted were the heel, fifth metatarsal, first metatarsal, sacrum, hip joint centre, and C7 vertebrae. A full description of the IMU data processing can be found in Supplementary Information.

Kinetic data from the insoles was also divided into strides and normalized to percent gait. Strides were separated using the right heel strike event, which was detected when the force measured by the right insole exceeded the cut-off value of 30 N. The IMU and insoles strides were aligned by synchronizing jump events that took place at the beginning of the trial. This synchronization allowed for correlations to be made between the changes in body kinematics and changes in ground reaction force behaviour.

Each stride was given a condition tag to indicate the ground conditions during that stride (Fig. 1F–H). As the ground conditions were uncontrolled and inconsistent in the winter, the winter trials were broken down into condition Winter 1 (W1) and Winter 2 (W2) where feasible. The W1 condition represented when the sidewalks had been sufficiently plowed and salted, such that the ground was predominantly free from ice and snow. The W2 condition was represented when the ground was predominantly covered in ice, snow, and slush. The Summer condition (S) was for all the strides in the summer data collection. The different periods of the ground conditions were determined from qualitative labelling of the videos taken during the experiments. During the winter experiments, the percentage of the strides that were during W1 and W2 were 61.3% ± 26.1% and 34.5% ± 27.0%, respectively. The occurrence of these three different conditions was dependent entirely on the natural conditions present during the experiment. There was one subject where no W2 conditions were present.

Two different measures of stability were used to observe the effects of the winter and summer conditions. The first stability measure was the MoS^3^ in both the AP and ML directions to quantify the relationship between the BoS and CoM. The ML MoS was measured as the minimum distance from the XCoM to the virtual fifth metatarsal marker of the stance foot during single stance where the XCoM is defined according to the following equation (Eq. 1):

1 $$\begin{aligned} \text {XCoM} = \text {CoM} + \sqrt{\frac{L}{g}}\dot{\text {CoM}} \end{aligned}$$

where *L* is the leg length of the inverted pendulum model (calculated for each stride as the average distance from the virtual foot marker to the CoM). and $\dot{\text {CoM}}$ is the CoM velocity. In the AP direction, two different values were calculated for the MoS, the forward MoS (FW MoS) and backward MoS (BW MoS). The FW MoS was calculated as AP distance between the virtual heel marker of the anterior foot and the XCoM at heel strike. The BW MoS was calculated as the AP distance between the virtual fifth metatarsal marker of the posterior foot at the same heel strike event. An increase in FW MoS suggests an increase in resistance to a forwards loss of stability, and an increase in the BW MoS suggests an increase in resistance to a backwards loss of stability^3,16^.

The second stability measure was the local divergence exponents (LDE) to provide a value that reflected the stability of the entire body^24^. The calculation of the LDE was conducted using a publicly available version^25^ of the algorithm suggested by Mehdizadeh^26^ with the embedding dimension set to 5^27^ and the delay set to 10^28^. The input data for the calculation was the normalized CoM velocity for 650 strides. A previous study has suggested that 150 strides is sufficient for calculating the LDE for gait^29^. However, since the terrain for the experiments was non-constant, we elected to include as many strides as possible for each subject while still ensuring that each subject used the same number of stride. Therefore, this analysis was only conducted on the entirety of the summer and winter datasets each, as there was not a sufficient number of strides for each condition of the winter conditions for all subjects.

To investigate possible changes in the contributions of the different stability strategies between the summer and winter experiments, two different multivariate linear regressions were created. The first regression investigated the dependency of the foot placement location at heel strike on the previous state of the CoM at midstance (Eq. 2).

2 $$\begin{aligned} {\begin{matrix} \Delta \text {FP} & = \beta _1\Delta \text {CoM}_x + \beta _2\Delta \text {CoM}_y + \beta _3\Delta \text {CoM}_z\\ & \quad + \beta _4\Delta \dot{\text {CoM}}_x + \beta _5\Delta \dot{\text {CoM}}_y + \beta _6\Delta \dot{\text {CoM}}_z\\ & \quad + \beta _7\Delta \ddot{\text {CoM}}_x + \beta _8\Delta \ddot{\text {CoM}}_y + \beta _9\Delta \ddot{\text {CoM}}_z \end{matrix}} \end{aligned}$$

where FP is the foot placement location, ($\beta _{1},...,\beta _{9}$) are the gains or regression coefficients, *x* refers to the AP direction, *y* the ML, and *z* the VT, and the delta symbol ($\Delta$) indicates the deviation from the average value of the term. Two regressions of this type were made, one for the AP direction and another for the ML direction. The gains of the independent variables ($\beta _{1},...,\beta _{9}$) and $R^2$ values of the regression were compared between the summer and winter experiments. A larger gain value suggested that there was a larger change in the stepping location for the same change in the CoM state, and a larger $R^2$ value suggested a greater adherence to the stepping strategy. One regression was created for the entire winter trial, as some subjects had very few strides for condition Winter 2. This regression has previously been applied to studies of indoor walking^7^.

In the ML direction, an additional regression was created to investigate if the ankle and trunk strategies compensated for errors made in step placement. The difference between the foot placement predicted by the previous regression and the actual foot placement was considered the error (Eq. 3). This error was then related to the change in the average ML CoP ($\Delta \overline{\text {CoP}}$) position and the average ML trunk angular velocity ($\Delta \overline{\omega }_\text {trunk}$) (Eq. 4).

3 $$\begin{aligned} \text {Error} = \Delta \text {FP}_\text {predicted} - \Delta \text {FP}_\text {actual} \end{aligned}$$

4 $$\begin{aligned} \text {Error} = \beta _\text {ankle}\Delta \overline{\text {CoP}} + \beta _\text {trunk} \Delta \overline{\omega }_\text {trunk} \end{aligned}$$

The $\text {CoP}$ position was determined from the instrumented insoles and the ${\omega }_\text {trunk}$ from the inertial measurement units (detailed in the supplemental material, all angular data has units of degrees). Similar to the previous foot placement regression, the gains of the independent variables ($\beta _\text {ankle}$ and $\beta _\text {trunk}$) and $R^2$ values of the regression were compared between the summer and winter experiments. A similar regression was previously conducted for a study of indoor walking but only for ankle recovery and not the trunk^30^.

Five different spatiotemporal measures were calculated to observe any stepping adjustments made when different ground conditions were encountered. We calculated stride time, stance time, speed, stride length, and step width. The mean values and root-mean-square (RMS) variability (intra-subject standard deviation) were compared for all measures. The stride time was computed as the time between successive right heel strikes, and the stance time was found as the time between right heel strike and right toe off. Speed was computed as the average AP CoM velocity during each stride. The stride length was computed as the AP distance between the right heel markers at the beginning and end of the stride. The step width was computed as the ML distance between the right and left heel markers at right and left heel strike. All gait events in this analysis were located using data from the IMU suit.

All measures were compared first between the entirety of the summer and winter experiments and then, if possible, subsequently compared between the S, W1, and W2 conditions. For results that could be further separated into W1 and W2 conditions, only those results have been shown in the main manuscript, and the remaining results from the summer and winter experiments can be found in Supplementary Information (Supplemental Table S1). Measures from the summer and winter experiments were compared using unpaired t-tests. To compare measures among the S, W1, and W2 conditions, a one-way ANOVA was conducted followed by post-hoc t-test with the Holm-Sidak correction for multiple comparisons^31^. If a result was significant, the effect size ($\eta ^2$) was calculated^32^. The significance value for all statistical tests was $\alpha = 0.05$. Prior to the statistical analysis, all data was nondimensionalized by leg length *L* and gravity *g*. Length was divided by *L* (mean 0.91 m), velocity by $\sqrt{gL}$ (mean 2.98 m/s), acceleration by *g* (9.806 m/s$^{2}$), and time by $\sqrt{L/g}$ (mean 0.30 s).

## Results

The stepping behaviour in the AP direction was the primary aspect that was affected by the summer and winter conditions. In the winter experiments as the conditions worsened from condition W1 to condition W2, subjects elected to walk with a greater average FW MoS and reduced AP MoS variability. Between the summer and winter experiments, the multiple gains of the AP stepping regression were also increased. In contrast, we found minimal changes in the ML direction (summarized in Table 1).

For the stability measures, the MoS and LDE were not significantly different between the summer and winter experiments in any of the three direction, with the exception of an increase in the BW MoS variability during the winter experiments ($p=0.024$, $\eta ^{2}=0.1690$) (Table 1, Supplemental Table S1, and Fig. 2B, respectively). However, additional differences were found when separating the winter data into W1 and W2 for the MoS measures. The FW MoS was increased by 23.5% from condition W1 compared to condition W2 ($p=0.0151$, $\eta ^{2}=0.1876$), and the FW MoS variability was the lowest in condition W2 compared to all other conditions ($p=5.583e{-}6$, $\eta ^{2}=0.4457$, Fig. 2A). As a result of an increases in the FW MoS, the BW MoS decreased by 23.5% from condition W1 to condition W2 ($p = 0.0015$, $\eta ^{2}=0.3087$), and similarly the variability of the BW MoS was the lowest compared to all other conditions ($p=0.0017$,$\eta ^{2}=0.2662$, Fig. 2A). The ML MoS was not significantly different among all three conditions (average $p=0.1527$ for mean and $p=0.1382$ for variability).

The stepping strategy regression showed differences in some gains between the winter and summer experiments, but the $R^2$ of the regressions were not significantly different. In the AP regression, differences were found for the AP position ($p=0.0207$, $\eta ^{2}=0.1706$), VT position ($p=0.0032$, $\eta ^{2}=0.2712$) and VT velocity ($p=0.0227$, $\eta ^{2}=0.1719$) coefficients (Fig. 3A). All significantly different gains were greater in the regression for the winter than the summer, suggesting a larger reaction to CoM state variation in the winter than the summer. Fewer differences were found in the ML regression (Fig. 3B), where only the AP position coefficient was different ($p=0.0167$, $\eta ^{2}=0.1878$). In both the AP and ML regressions, the $R^2$ values were not significantly different ($p=0.1816$ and $p=0.2042$, AP and ML respectively) between the experiments.

In the additional strategy regression to capture the contributions of the ankle and trunk strategies (Fig. 4), only the trunk coefficients were statistically significant from zero in both the summer and winter experiments ($p=1.191e{-}6$ and $p=0.0001$, summer and winter respectively). However, the values of the coefficients ($p=0.7406$ and $p=0.8322$, ankle and trunk respectively) and the $R^2$ ($p=0.6570$) values of the regressions were not significantly different between the summer and winter experiments.

Few changes were found for the spatiotemporal measures (Table 2). Mean stride time, walking speed, stride length, step width, and their RMS variabilities did not change significantly. Mean stance time also did not change. Only stance time variability changed and was decreased by an average of 26.7% in condition W2 compared to all other conditions ($p=0.0051$, $\eta ^{2}=0.2269$).

## Discussion

We investigated how gait behaviour would be affected by walking conditions in the summer and winter, with a focus on stability. We expected to find changes in both stability and spatiotemporal measures due to reduced possibilities for adequate foot placement locations during winter conditions. At the same time, we also expected the average ML MoS to remain constant and the average BW MoS to decrease, while the variability of both the ML and AP MoS (FW and BW MoS) increased. We expected that the changes in the MoS would coincide with changes in the related spatiotemporal measures, specifically decreased stride length, increased step width, and increased variability of both step width and stride length. In agreement with our hypothesis we did find that the average ML MoS did not significantly vary with the ground condition. However, contrary to our hypothesis, the variability of the ML MoS was also unaffected by the change in ground condition (Fig. 2B and Table 1). In the AP direction, we found that when walking on more difficult terrain, the average FW MoS increased and the BW MoS decreased, favouring a resistance to a loss of forwards stability. The variability of the FW and BW MoS also decreased. Similarly, the spatiotemporal results also disagreed with our hypothesis with no significant differences found in the average value or variability of the stride length and step width (Table 2). The results of the stepping regression do not suggest that the stepping strategy was hindered by ice and snow, as evident by the similar $R^2$ values of the stepping regressions in the AP and ML directions (Fig. 3). However, the gains of the AP stepping regression were increased in the AP and VT direction during the winter experiments, whereas no meaningful changes were observed in the ML stepping regression (Fig. 3). Similarly, findings from the regression for the ML ankle and trunk strategy (Fig. 4) suggests that there were no additional contributions from the ankle or trunk strategy in the winter experiments compared to walking in the summer.

The increase in the FW MoS and the simultaneous decrease in the BW MoS, favouring a resistance to a forward loss of stability, when walking in the W2 condition (Fig. 2B) contradicts the findings of a previous study that exposed subjects to known slippery conditions^16^. One key difference between the previous study and the current study is that in the previous study the subjects were subjected to identical slip conditions repetitively, whereas in our study the friction of the ground surface was likely different from stride to stride. As a result, our subjects may have been uncertain as to whether the friction between the foot and the ground would be able to supply a sufficient braking force to stop a forward loss of balance. This behaviour is loosely shown in the vertical ground reaction forces (Supplemental Fig. S3), where the mean peak ground reaction force during heel strike was 5.86% and 5.10% lower for W2 than for S and W1, respectively. While this decreased force was not statistically significant ($p=0.1177$), it suggests that the some of the subjects may have been hesitant to trust the slippery surface at initial contact. In contrast, there was no discernible difference in the peak ground reaction force among the three conditions at push-off, suggesting that once subjects made contact and did not slip, they applied a larger force at push-off. This hesitancy of trusting the braking force is further observed in the body kinematics in which the range of the AP CoM velocity and acceleration (Supplemental Fig. S1) and the trunk angular velocity (Supplemental Fig. S2) are significantly decreased in condition W2. By decreasing the range of the AP CoM velocity, the anterior position of the XCoM at initial contact was reduced, increasing the MoS while allowing stride length to remain constant (Table 2). This change in body kinematics allowed for the FW MoS to be increased when walking in more treacherous conditions, providing a larger resistance to the forward losses of stability that may occur due to a lack of braking force from reduced friction. Conversely, the increase in the FW MoS resulted in a decrease in the BW MoS, reducing the resistance to a backwards loss of balance, which may result in a higher likelihood of falling backwards.

The stepping strategy regressions (Fig. 3) revealed that stepping strategy was not hindered by the ice and snow present in the winter experiments. A previous study on walking with external stabilization found that when walking was stabilized in the ML direction, the $R^2$ value of a regression between the foot placement and CoM position and velocity was decreased, suggesting a decrease in the stepping control of walking^33^. Our $R^2$ values were not significantly different in either the AP or ML direction, suggesting that there was a similar contribution of the stepping strategy during the summer and winter experiments. In the AP direction, there were significant differences in the values of the gains for the AP position (*x*), VT position (*z*), and VT velocity ($\dot{z}$). For each of these three gains, the value was higher in the winter experiment, suggesting a larger reaction in foot placement to a variation in the state of the CoM. One of the surprising results is that the value of the gains in the VT direction were significantly different from zero for the AP regression, which has not been observed in previous studies^7^. A relevant difference between this study and the previous studies is that this study was not conducted on level, flat ground (Fig. 1E). Slope variations of the walking surface may have caused larger variations in the VT state of the CoM that affected the foot placement. Unlike the AP stepping regression, the ML stepping regression was not largely affected by the winter conditions, contrary to the hypothesis that stepping would be altered. The only change present in the regression was the gain of the AP position. The value of the gain for the AP position independent variable was fairly low compared to the gains of the other independent variables and only the AP position gain in the summer was statistically different from zero (Supplemental Table S3), leading us to conclude that the ML stepping regression is not largely affected by the summer and winter experiments at least in our study.

The ankle and trunk strategy regression (Fig. 4) showed no additional compensation from the ankle and trunk strategy during the winter experiment. The $R^2$ values of the fits were relatively low compared to the stepping regressions. The gain for the trunk strategy was significantly different from zero but not significantly different between summer and winter experiments. The ankle strategy gain, however, was not significantly different from zero. These results suggest that the ML trunk motion was affected by the errors in foot placement whereas the ML CoP was not. This contradicts the previous findings of van Leeuwen et al.^30^ that found the mean value of the CoP displayed a negative correlation with the error made in the prior step. Possible causes of this difference may be due to the ground conditions not allowing for accurate control of the CoP due to uneven surfaces. Additionally, a previous study^34^ using similar insoles found that the estimation of the range and standard deviation of the ML CoP is lower in the insole measurements compared to the in-ground force plates such as from van Leeuwen et al. The significant values of the trunk gain do suggest that the trunk strategy may be used to compensate for inaccuracies in the foot placement. However, it is not possible to discern whether the relationship between the error in foot placement and change in trunk angular velocity is due to active compensation from the trunk strategy or passive dynamics. It is possible that the change in the angular velocity of the trunk is a consequence of foot placement inaccuracy rather than a corrective action.

There are some limitations of the current study that must be considered. As a result of the unstructured environments in which the experiments took place, it is challenging to entirely discern the factors that may have led to changes in behaviour. It was assumed that the primary contributor to changes in gait was the presence of snow and ice in the winter experiments. However, it is also possible that other factors such as changes in pedestrians traffic, environmental noise^35^, ambient temperature^36^, or practical changes in clothing contributed to the change in observed behaviour. We believe the change in footwear is a limited contributing factor to some of the observed changes with changes primarily found in gait kinematics than stability measures^37^. Additionally, we believe that temperature can impact gait behaviour, affecting subject comfort and metabolic cost for example. While outside the scope of this study, future investigations could include control of clothing and detailed recordings of weather conditions (such as wind chill) to determine the effect of temperature and thermodynamics on gait. The current study also only included young healthy adults, for which the tested conditions may not have presented much of a challenge to their gait stability in comparison to other populations such as older adults. Additionally, the equipment used in the data collection, IMU-based motion capture and instrumented insoles, are known to be less accurate than their traditional counterparts, optical motion capture and in-ground force plates^22,34^. One of our previous studies compared stability measures derived from the IMU suit and optical motion capture^38^. We found that although the accuracy of the IMU system may be reduced, they are still sensitive to changes in behaviour, allowing for meaningful insights to be gained. The different ground conditions (S, W1, and W2) were also determined based on qualitative labelling performed by a single individual which might differ slightly if conducted by a different individual.

There are several meaningful conclusions that can be drawn from our study results. When traversing winter ground conditions, stability in the walking direction is maintained through adaptations in the foot placement, increasing the AP MoS and sensitivity to change in CoM state. Conversely, the snowy and icy conditions do not have a significant effect on ML stability and thus no additional compensation required from the ankle and trunk strategies, at least for young, healthy adults. These results provide further insight that even in outdoor conditions, stepping is the primary method used to maintain stability, regardless of whether there are ice and snow present. Therefore, rehabilitation focused on the stepping strategy could aid reducing the incidence of falls exacerbated by hazardous outdoor conditions.

## Supplementary Information

Supplementary Information.

## Data Availability

The data used in the current study are available in Queen’s University Dataverse repository at 10.5683/SP3/EY9PAJ.

## References

[1] Morency P, Voyer C, Burrows S, Goudreau S (2012). Outdoor falls in an urban context: Winter weather impacts and geographical variations. Can. J. Public Health.

[2] Bergland A, Jarnlo GB, Laake K (2013). Predictors of falls in the elderly by location. Aging Clin. Exp. Res..

[3] Hof AL, Gazendam MG, Sinke WE (2005). The condition for dynamic stability. J. Biomech..

[4] Horak FB (2006). Postural orientation and equilibrium: What do we need to know about neural control of balance to prevent falls?. Age Ageing.

[5] Bruijn SM, Van Dieën JH (2018). Control of human gait stability through foot placement. J. R. Soc. Interface.

[6] Winter DA (1995). Human balance and posture control during standing and walking. Gait Posture.

[7] Wang Y, Srinivasan M (2014). Stepping in the direction of the fall: The next foot placement can be predicted from current upper body state in steady-state walking. Biol. Lett..

[8] Hof AL, Vermerris SM, Gjaltema WA (2010). Balance responses to lateral perturbations in human treadmill walking. J. Exp. Biol..

[9] Vlutters M, Van Asseldonk EH, Van Der Kooij H (2016). Center of mass velocity-based predictions in balance recovery following pelvis perturbations during human walking. J. Exp. Biol..

[10] Vlutters M, van Asseldonk EH, van der Kooij H (2018). Reduced center of pressure modulation elicits foot placement adjustments, but no additional trunk motion during anteroposterior-perturbed walking. J. Biomech..

[11] Best AN, Wu AR (2020). Upper body and ankle strategies compensate for reduced lateral stability at very slow walking speeds. Proc. R. Soc. B Biol. Sci..

[12] van den Bogaart M, Bruijn SM, Spildooren J, van Dieën JH, Meyns P (2022). The effect of constraining mediolateral ankle moments and foot placement on the use of the counter-rotation mechanism during walking. J. Biomech..

[13] Voloshina AS, Kuo AD, Daley MA, Ferris DP (2013). Biomechanics and energetics of walking on uneven terrain. J. Exp. Biol..

[14] Curtze C, Hof AL, Postema K, Otten B (2011). Over rough and smooth: Amputee gait on an irregular surface. Gait Posture.

[15] Gates DH, Scott SJ, Wilken JM, Dingwell JB (2013). Frontal plane dynamic margins of stability in individuals with and without transtibial amputation walking on a loose rock surface. Gait Posture.

[16] Bone MD (2021). Investigating proactive balance control in individuals with incomplete spinal cord injury while walking on a known slippery surface. Neurosci. Lett..

[17] Matthis JS, Yates JL, Hayhoe MM (2018). Gaze and the control of foot placement when walking in natural terrain. Curr. Biol..

[18] Kowalsky DB, Rebula JR, Ojeda LV, Adamczyk PG, Kuo AD (2021). Human walking in the real world: Interactions between terrain type, gait parameters, and energy expenditure. PLoS ONE.

[19] Schmitt AC (2021). Walking indoors, outdoors, and on a treadmill: Gait differences in healthy young and older adults. Gait Posture.

[20] Kingston, O. N. 7 Day Forecast—Environment Canada (2020).

[21] Xsens Technologies B.V. MVN User Manual (2020).

[22] Mavor MP, Ross GB, Clouthier AL, Karakolis T, Graham RB (2020). Validation of an IMU suit for military-based tasks. Sensors.

[23] Schepers, M., Giuberti, M. & Bellusci, G. Xsens MVN: Consistent tracking of human motion using inertial sensing. *Xsens Technologies B.V.* 1–8 (2018).

[24] Bruijn SM, Meijer OG, Beek PJ, Van Dieen JH (2013). Assessing the stability of human locomotion: A review of current measures. J. R. Soc. Interface.

[25] Bruijn, S. SjoerdBruijn/LocalDynamicStability: First release, 10.5281/ZENODO.573285 (2017).

[26] Mehdizadeh S (2019). A robust method to estimate the largest Lyapunov exponent of noisy signals: A revision to the Rosenstein’s algorithm. J. Biomech..

[27] Dingwell JB, Cusumano JP (2000). Nonlinear time series analysis of normal and pathological human walking. Chaos.

[28] England SA, Granata KP (2007). The influence of gait speed on local dynamic stability of walking. Gait Posture.

[29] Bruijn SM, Van Dieën JH, Meijer OG, Beek PJ (2009). Statistical precision and sensitivity of measures of dynamic gait stability. J. Neurosci. Methods.

[30] van Leeuwen AM, van Dieën JH, Daffertshofer A, Bruijn SM (2021). Ankle muscles drive mediolateral center of pressure control to ensure stable steady state gait. Sci. Rep..

[31] Cardillo, G. Holm-Sidak t-test - File Exchange - MATLAB Central (2006).

[32] Hentschke, H. hhentschke/measures-of-effect-size-toolbox https://github.com/hhentschke/measures-of-effect-size-toolbox (2023).

[33] Mahaki M, Bruijn SM, Van Dieën JH (2019). The effect of external lateral stabilization on the use of foot placement to control mediolateral stability in walking and running. PeerJ.

[34] Oerbekke MS (2017). Concurrent validity and reliability of wireless instrumented insoles measuring postural balance and temporal gait parameters. Gait Posture.

[35] Franek M, Režný L, Šefara D, Cabal J (2018). Effect of traffic noise and relaxations sounds on pedestrian walking speed. Int. J. Environ. Res. Public Health.

[36] Obuchi SP, Kawai H, Garbalosa JC, Nishida K, Murakawa K (2021). Walking is regulated by environmental temperature. Sci. Rep..

[37] Garrah, S. N., Best, A. N. & Wu, A. R. Changes in seasonal footwear elicited alterations in gait kinematics but not stability. *bioRxiv*10.1101/2023.05.18.541138.

[38] Riek PM, Best AN, Wu AR (2023). Validation of inertial sensors to evaluate gait stability. Sensors.

