# Supplementary material for: Modified stepping behaviour during outdoor winter walking increases resistance to forward losses of stability
Source: Sci Rep. 2023 May 24;13:8432. doi: 10.1038/s41598-023-34831-3 (PMC10209205; doi:10.1038/s41598-023-34831-3)
Supplement: Supplementary file 1 — Supplementary Information. [file 41598_2023_34831_MOESM1_ESM.pdf]

Supplementary Information

Modified stepping behaviour during outdoor winter walking increases resistance to forward losses of stability

Aaron N. Best[1,*] **and Amy R. Wu**[1]

[1]Queen's University, Ingenuity Labs Research Institute, Mechanical and Materials Engineering, Kingston, K7L 2N9, Canada
[*]aaron.best@queensu.ca

Introduction

This document serves to provide additional results and details to support the main manuscript. The following sections of the supplemental material will provide additional detail on the methods used to create the publicly available dataset, additional results on basic kinematic and kinetic behaviour, and full results produced by statistical comparisons of all measures in the main text and supplementary material. The sections of this document are:

1. IMU Data Processing
2. CoM Kinematics
3. Trunk Kinematics
4. Foot Clearance
5. Ground Reaction Force
6. Centre of Pressure
7. Results Summary Tables

IMU Data Processing

The kinematic data from the IMU suit was divided into strides (819.6 ± 66.9 strides per experiment on average) and normalized to percent gait delineated by right heel strike to right heel strike. The heel strike and toe off gait events used to separate this data were found using the contact detection provided by the Xsens MVN software. The data provided by the Xsens MVN software included the position (O_{ANAT}) and orientation ($[x^{3\times1}_{\text{ANAT}}, y^{3\times1}_{\text{ANAT}}, z^{3\times1}_{\text{ANAT}}]$) of the local coordinate systems for each of the tracked segments. Additionally, the data included the local coordinates of markers that estimate the location of anatomical features within each segment (P_{ANAT}). The global location (P_{GLOBAL}) of required markers was obtained by using the provided orientation and position of each local coordinate system to create a transformation matrix that transported the local points into the global coordinate system for the experiment (Equation 1). The required markers that were extracted were the heel, fifth metatarsal, first metatarsal, sacrum, hip joint centre, and C7 vertebrae. The Xsens software also provided an estimate of the body CoM in the global coordinate system.

$$\begin{Bmatrix} P^X_{\text{GLOBAL}} \\ P^Y_{\text{GLOBAL}} \\ P^Z_{\text{GLOBAL}} \\ 1 \end{Bmatrix} = \begin{bmatrix} x^X_{\text{ANAT}} & y^X_{\text{ANAT}} & z^X_{\text{ANAT}} & O^X_{\text{ANAT}} \\ x^Y_{\text{ANAT}} & y^Y_{\text{ANAT}} & z^Y_{\text{ANAT}} & O^Y_{\text{ANAT}} \\ x^Z_{\text{ANAT}} & y^Z_{\text{ANAT}} & z^Z_{\text{ANAT}} & O^Z_{\text{ANAT}} \\ 0 & 0 & 0 & 1 \end{bmatrix} \begin{Bmatrix} P^x_{\text{ANAT}} \\ P^y_{\text{ANAT}} \\ P^z_{\text{ANAT}} \\ 1 \end{Bmatrix} \tag{1}$$

To allow the recorded motions to be resolved into the anatomical directions (AP, ML, and VT), a single coordinate system was created for each stride, which we refer to as the stride coordinate system. The x axis ($x^{3\times1}_{\text{STRIDE}}$) of the stride coordinate system was a unit vector that began at the CoM at the start of the stride (O_{STRIDE}) and pointed towards the CoM position at the end of the stride; this axis was taken as the AP direction. The y axis ($y^{3\times1}_{\text{STRIDE}}$) (ML direction) was taken as the cross product between the global z axis and the stride coordinate system x axis. Finally, the z axis ($z^{3\times1}_{\text{STRIDE}}$) was taken as the cross product between the x and y axis of the stride coordinate system. The various markers that were previously placed into the global coordinate system were then transformed into the stride coordinate (P_{STRIDE}) system (Equation 2) to allow further analysis.

$$\begin{Bmatrix} P^{x}_{\mathrm{STRIDE}} \\ P^{y}_{\mathrm{STRIDE}} \\ P^{z}_{\mathrm{STRIDE}} \\ 1 \end{Bmatrix} = \begin{bmatrix} x^{X}_{\mathrm{STRIDE}} & y^{X}_{\mathrm{STRIDE}} & z^{X}_{\mathrm{STRIDE}} & O^{X}_{\mathrm{STRIDE}} \\ x^{Y}_{\mathrm{STRIDE}} & y^{Y}_{\mathrm{STRIDE}} & z^{Y}_{\mathrm{STRIDE}} & O^{Y}_{\mathrm{STRIDE}} \\ x^{Z}_{\mathrm{STRIDE}} & y^{Z}_{\mathrm{STRIDE}} & z^{Z}_{\mathrm{STRIDE}} & O^{Z}_{\mathrm{STRIDE}} \\ 0 & 0 & 0 & 1 \end{bmatrix}^{-1} \begin{Bmatrix} P^{X}_{\mathrm{GLOBAL}} \\ P^{Y}_{\mathrm{GLOBAL}} \\ P^{Z}_{\mathrm{GLOBAL}} \\ 1 \end{Bmatrix} \tag{2}$$

CoM Kinematics

The variation in the kinematics of the body CoM were analyzed to investigate the effect of the different conditions on the entire body motion. To determine if there was a significant difference in the behaviour of the CoM, the range of the CoM position, velocity, and acceleration were compared in all three directions. There were some observed changes in the CoM kinematics (Figure S1 and Table S2). The range of the AP CoM velocity ($p = 0.0022$) and acceleration ($p = 0.0155$) was decreased from condition W1 to condition W2. The range of the CoM acceleration was decreased in all three directions from condition W1 to condition S ($p = 0.0129$, $p = 0.0058$, and $p = 0.0134$, AP, ML, and VT respectively).

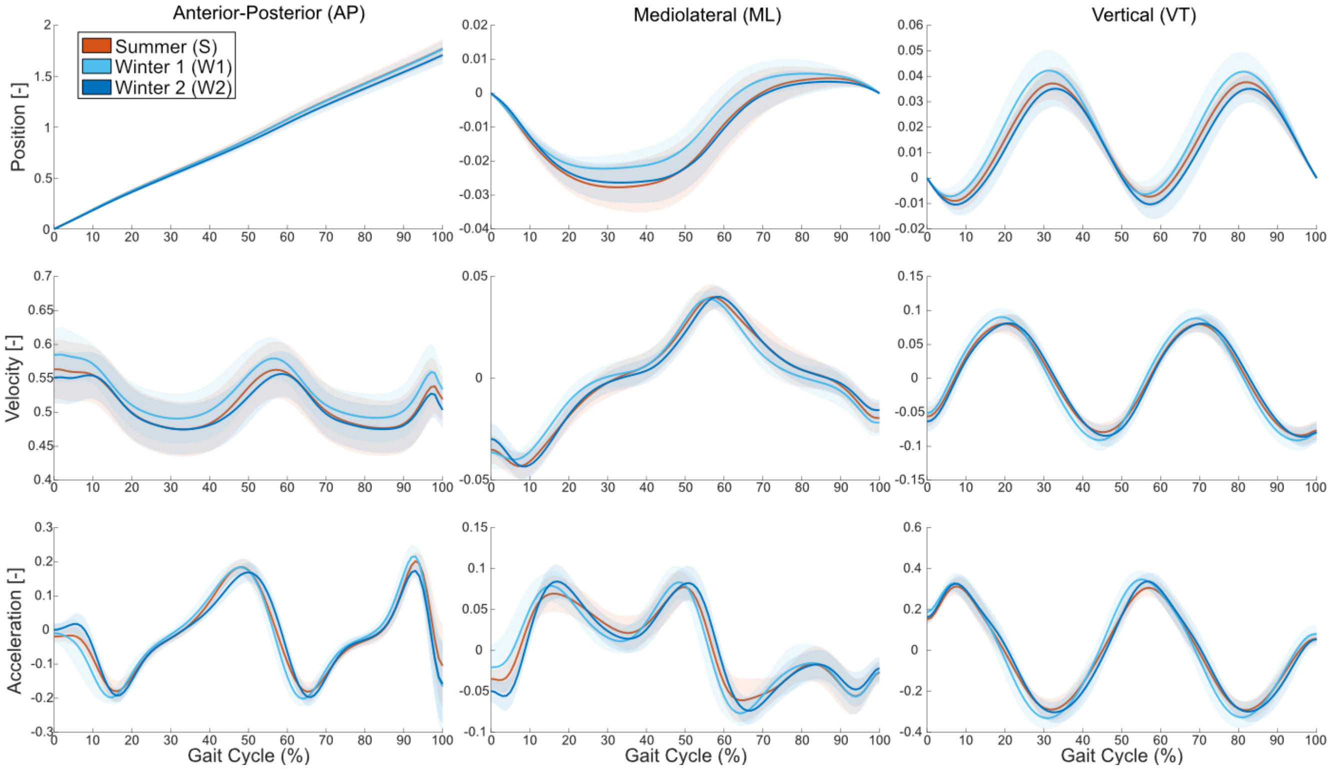

Figure S1. Body centre of mass (CoM) position, velocity, and acceleration for the condition S ($N = 15$), condition W1 ($N = 15$), and condition W2 ($N = 14$). The mean trajectories across all subjects (solid lines) and one s.d. (shaded region) are shown for each condition in dimensionless form as a percentage of gait cycle. Positive values are in the forward (AP), left (ML), and upwards (VT) directions.

Trunk Kinematics

The angular trunk kinematics were also compared between the different conditions. The AP and ML trunk angles were calculated as the angle between the line joining the sacrum and C7 markers and the z axis of the stride coordinate system in the AP and ML directions respectively. The ranges of the trunk angles and their first derivatives and the mean AP trunk angle were compared. All angles and angular velocities were calculated in degrees prior to being normalized. There were minor changes in the trunk angle kinematics from the different conditions (Figure S2 and Table S2) with the range of the AP trunk angular velocity decreased by 33.3% or 9.87e-4$\frac{\circ}{s}$ from condition W1 to condition W2 ($p = 0.0037$).

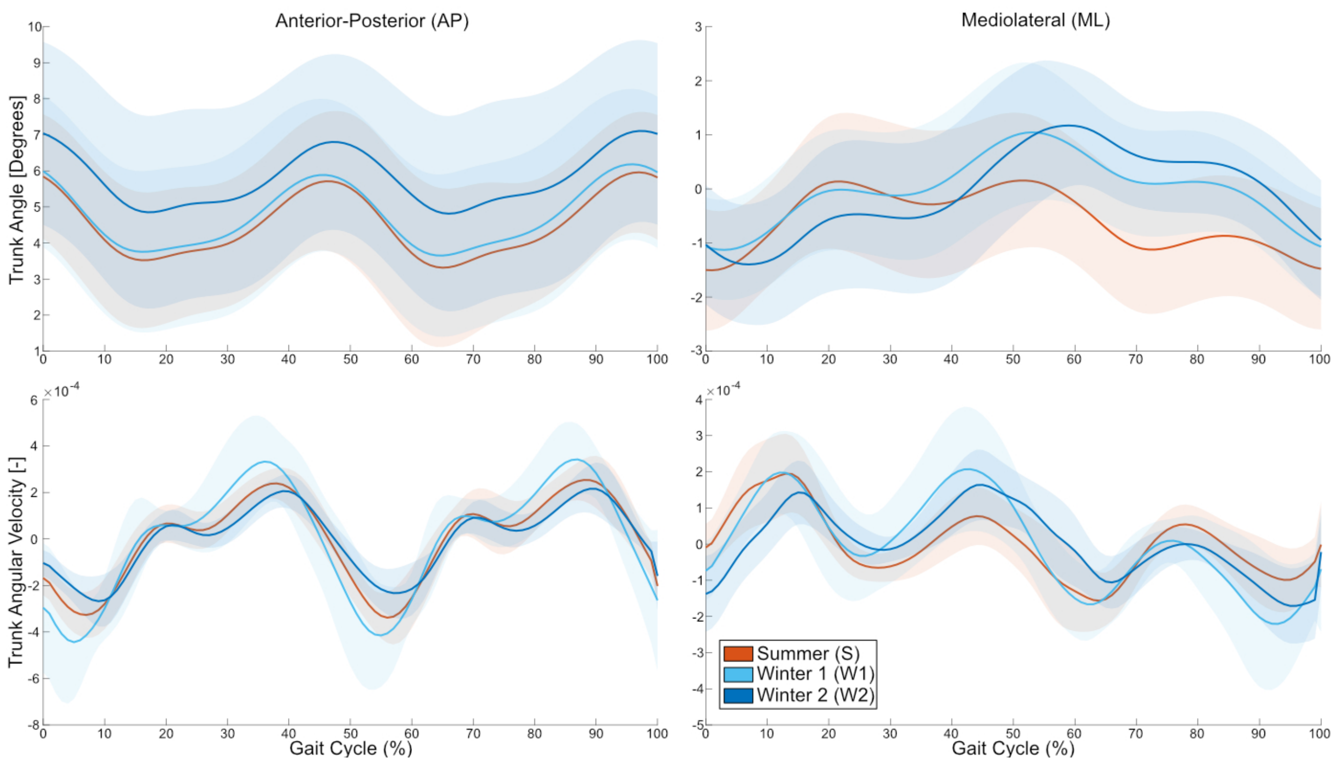

Figure S2. Trunk angle and angular velocity in the AP and ML direction for condition S ($N = 15$), condition W1 ($N = 15$), and condition W2 ($N = 14$). The mean trajectories across all subjects (solid lines) and one s.d. (shaded region) are shown for each condition in degrees (angle) and in dimensionless form (angular velocity) as a percentage of gait cycle. Positive values indicate forward (AP) and rightward (ML) trunk lean.

Foot Clearance

To determine if subjects altered the vertical foot clearance when walking in different conditions, we calculated a virtual ground clearance measure using the method described by Kowalsky et al.[1]. In this analysis, a virtual foot marker was defined as the average between the heel marker and the midpoint between the fifth and first metatarsal markers. In each step, a virtual ground was created as a line that joined the location of the foot marker at the beginning and end of the step. The virtual ground clearance was then calculated as the perpendicular distance between the virtual ground and foot marker. The minimum and maximum values of the virtual ground clearance were compared, and no significant differences were found between the conditions (Table S2).

Ground Reaction Force

Using the data from the instrumented insoles, we found that on average the peak ground reaction force during heel strike was lower for condition W2 (mean peak force in body weight: 1.06 ± 0.06 (S), 1.05 ± 0.07 (W1), and 0.99 ± 0.07 (W2)) but this result was not statistically significant ($p = 0.1177$, Table S2). Although this result was not significant, it does suggest that the subjects may have been hesitant to trust the slippery surface at initial contact. Comparing this peak heel strike force to the peak force at push off, there were no discernible differences between the conditions (mean peak force in body weight: 1.04 ± 0.12 (S), 1.03 ± 0.10 (W1), and 1.03 ± 0.11 (W2), $p = 0.9241$). Although not significant in our dataset, the reduced heel strike force and similar push off force in condition W2 compared to the other conditions suggest that the subjects chose to reduce the ground reaction force at initial contact but once they made contact and did not slip they applied a larger force at push-off. Due to insole equipment failure, all 15 subjects from the summer data collection but only 9 of the 15 winter subjects were included in this analysis.

Centre of Pressure

We assessed how the CoP behaviour changed in order to investigate changes in the application of the ankle strategy when walking in the different conditions. The primary data that was used from the instrumented insoles was the CoP in the AP

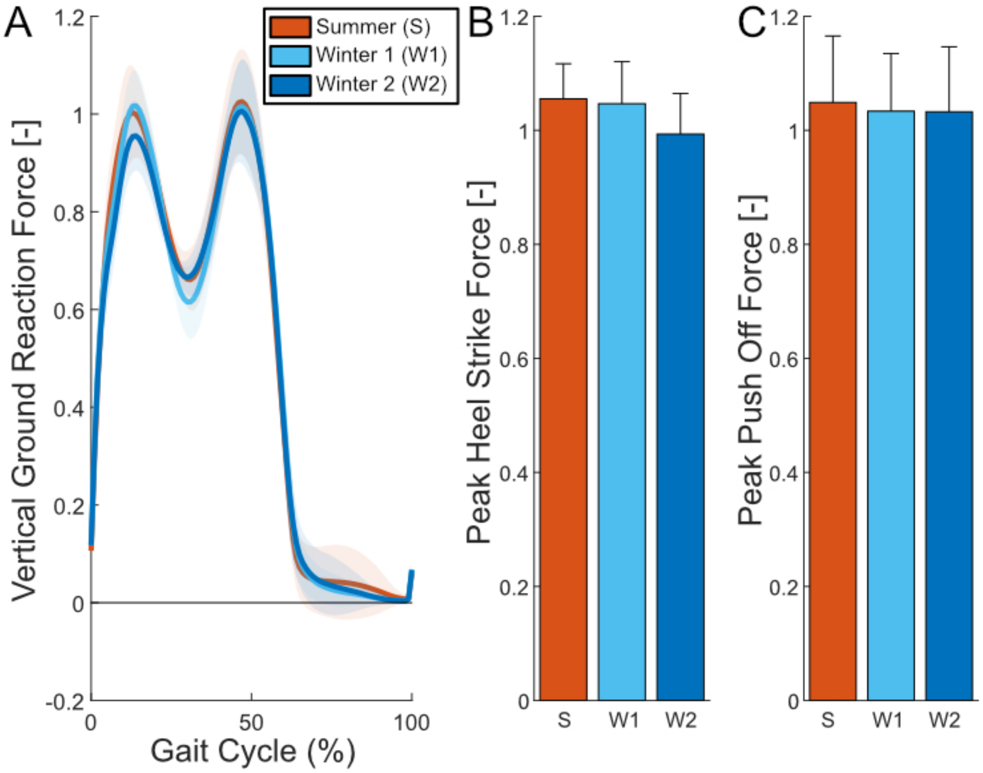

Figure S3. Vertical ground reaction forces for condition S ($N = 15$), condition W1 ($N = 9$), and condition W2 ($N = 8$). (A) Mean force trajectories(solid) and one s.d. (shaded region) shown in dimensionless form by gait cycle for the three different conditions. Mean and standard deviation of the peak force during (B) heel strike and (C) push-off for the three conditions.

and ML direction. The range of both the AP and ML CoP during stance and the mean value of ML CoP during stance were compared between the different conditions. Similar to the ground reaction forces analysis, all 15 subjects from the summer data collection, and 9 of the 15 winter subjects with insole data were included. The range of the AP ($p = 0.7035$) and ML ($p = 0.9066$) CoP was unaffected by the different conditions (Figure S4). The mean value of the ML CoP was determined to be significantly different among the three conditions ($p = 0.0442$), but the post hoc t-tests did not reveal any significant differences.

Results Summary Tables

This section contains tables that summarize all the measures and statistical comparisons of the current study. Table S1 details the comparison of all measures for the entirety of the summer and winter trials. Table S2 summarizes the comparison of all the measures between the Summer (S), Winter 1 (W1), and Winter 2 (W2) conditions. Table S3 summarizes the values and statistics relevant to the stepping regressions. Table S4 summarizes the values and statistics relevant to the ankle and trunk recovery regression. All measures shown have been non-dimensionalized.

References

1. Kowalsky, D. B., Rebula, J. R., Ojeda, L. V., Adamczyk, P. G. & Kuo, A. D. Human walking in the real world: Interactions between terrain type, gait parameters, and energy expenditure. *PLOS ONE* **16**, e0228682, DOI: 10.1371/JOURNAL.PONE.0228682 (2021).

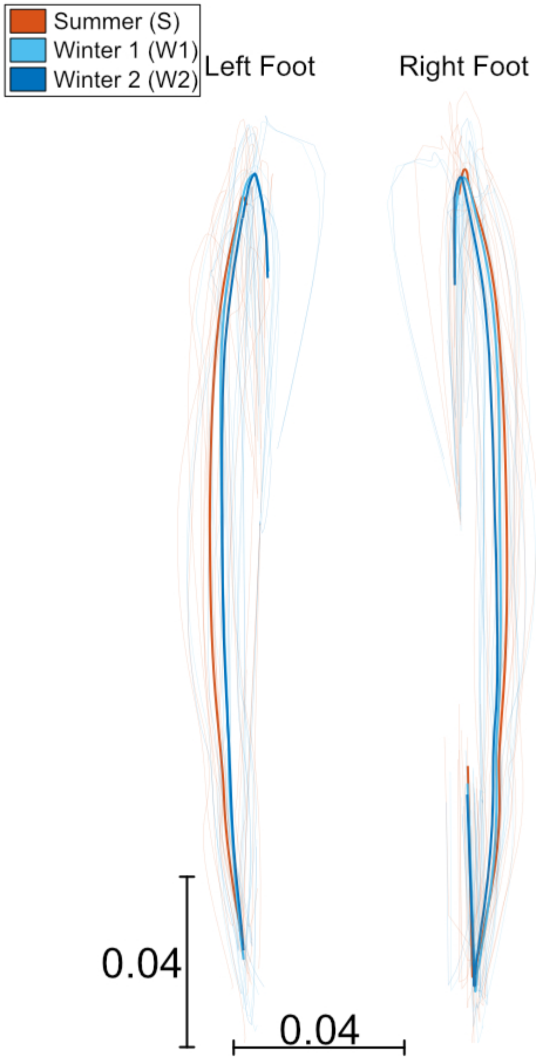

Figure S4. Trajectories of the CoP in condition S ($N = 15$), condition W1 ($N = 9$), and condition W2 ($N = 8$) conditions. Both the mean trajectories across all subjects (bold lines) and each subject's mean trajectory (faded lines) are shown for each condition.

Measure	Summer	Winter	P	η^2
AP LDE	2.0249 ± 0.1633	2.0488 ± 0.1601	0.6881	
ML LDE	1.8651 ± 0.1036	1.9229 ± 0.1157	0.1600	
VT LDE	1.7788 ± 0.0847	1.8184 ± 0.0897	0.2251	
FW MoS Mean	-0.1349 ± 0.0418	-0.1516 ± 0.0352	0.2463	
BW MoS Mean	0.8484 ± 0.0683	0.8511 ± 0.0524	0.9037	
ML MoS Mean	0.0428 ± 0.021	0.0295 ± 0.0213	0.0945	
FW MoS Variability	0.0478 ± 0.0094	0.0487 ± 0.0098	0.8084	
BW MoS Variability	0.0687 ± 0.0112	0.0800 ± 0.0524	0.0240*	0.1690
ML MoS Variability	0.0218 ± 0.0057	0.025 ± 0.004	0.0794	
AP CoM Position Range	1.7703 ± 0.0945	1.7492 ± 0.0809	0.5174	
ML CoM Position Range	0.034 ± 0.0072	0.0303 ± 0.0048	0.1121	
VT CoM Position Range	0.0487 ± 0.0074	0.0513 ± 0.0075	0.3347	
AP CoM Velocity Range	0.0944 ± 0.0099	0.0929 ± 0.0097	0.6807	
ML CoM Velocity Range	0.0845 ± 0.0116	0.0815 ± 0.0081	0.4225	
VT CoM Velocity Range	0.1692 ± 0.0271	0.1854 ± 0.0278	0.1176	
AP CoM Acceleration Range	0.4186 ± 0.0597	0.4494 ± 0.0493	0.1357	
ML CoM Acceleration Range	0.1639 ± 0.0177	0.1783 ± 0.0213	0.0525	
VT CoM Acceleration Range	0.6275 ± 0.0867	0.6976 ± 0.0928	0.0414*	0.1403
AP Trunk Angle Range	3.0408 ± 0.7662	2.8114 ± 0.6077	0.3713	
ML Trunk Angle Range	2.482 ± 0.7381	2.6663 ± 1.1141	0.5974	
AP Trunk Omega Range	0.0004 ± 0.0001	0.0004 ± 0.0002	0.9987	
ML Trunk Omega Range	0.0004 ± 0.0001	0.0004 ± 0.0001	0.7807	
Mean AP Trunk Angle	4.5042 ± 1.8298	5.2228 ± 2.2878	0.3502	
Stride Time	3.5145 ± 0.2253	3.4106 ± 0.2322	0.2239	
Stance Time	2.1198 ± 0.1546	2.0533 ± 0.1551	0.2494	
Speed	0.5128 ± 0.041	0.5219 ± 0.0335	0.5105	
Stride Length	1.7711 ± 0.0945	1.7501 ± 0.0808	0.5177	
Step Width	0.0988 ± 0.0457	0.0885 ± 0.0517	0.5684	
Stride Time Variability	0.0802 ± 0.0169	0.0882 ± 0.0233	0.2913	
Stance Time Variability	0.0987 ± 0.0214	0.1024 ± 0.0221	0.6445	
Speed Variability	0.0214 ± 0.0041	0.0249 ± 0.0056	0.0644	
Stride Length Variability	0.0601 ± 0.0097	0.0741 ± 0.0234	0.0422*	0.1393
Step Width Variability	0.0346 ± 0.0037	0.0377 ± 0.0062	0.1002	
Min Clearance Mean	0.002 ± 0.0008	0.0022 ± 0.0015	0.6510	
Max Clearance Mean	0.1838 ± 0.0152	0.1862 ± 0.0187	0.7027	
Min Clearance Variability	0.0032 ± 0.0012	0.0041 ± 0.0024	0.2348	
Max Clearance Variability	0.0104 ± 0.0014	0.0141 ± 0.0034	0.0005*	0.3539
AP CoP Range	0.1858 ± 0.0158	0.1889 ± 0.0122	0.4778	
ML CoP Range	0.0129 ± 0.0038	0.0134 ± 0.0055	0.7080	
ML CoP Peak	0.0118 ± 0.0035	0.0114 ± 0.0034	0.6781	
ML CoP Mean	0.0068 ± 0.0025	0.0055 ± 0.0025	0.0927	

Table S1. Summary table of all measures compared between the entirety of the summer and winter trials (N = 15). Values in the summer and winter represent the subject mean and standard deviation. The P column represents the p-value produced from a one-way ANOVA ($*p < 0.05$). The η^2 column represents the effect size calculated for statistically significant results.

Measure	Summer (S)	Winter 1 (W1)	Winter 2 (W2)	P	η^2	t-test (S and W1)	η^2	t-test (S and W2)	η^2	t-test (W1 and W2)	η^2
FW MoS Mean	-0.1349 ± 0.0418	-0.1706 ± 0.0479	-0.13 ± 0.0389	0.02801*	0.1600	0.0287		0.7591		0.0151*	0.1876
BW MoS Mean	0.8484 ± 0.0683	0.8852 ± 0.0722	0.8018 ± 0.0552	0.0060*	0.2209	0.1343		0.0640		0.0015*	0.3087
ML MoS Mean	0.0428 ± 0.021	0.0275 ± 0.0218	0.0321 ± 0.0223	0.1527							
FW MoS Variability	0.0478 ± 0.0094	0.0458 ± 0.0084	0.0314 ± 0.0071	5.5835e-06*	0.4457	0.5142		4.38e-06*	0.5088	3.49e-05*	0.4808
BW MoS Variability	0.0687 ± 0.0112	0.0733 ± 0.0116	0.0561 ± 0.0144	0.0018*	0.2662	0.3176		0.009*	0.2070	5.67e-4*	0.3206
ML MoS Variability	0.0218 ± 0.0057	0.0239 ± 0.0037	0.0255 ± 0.005	0.1382							
AP CoM Position Range	1.7703 ± 0.0945	1.7672 ± 0.0786	1.7091 ± 0.0825	0.1102							
ML CoM Position Range	0.034 ± 0.0072	0.0299 ± 0.0047	0.0315 ± 0.0052	0.1610							
VT CoM Position Range	0.0487 ± 0.0074	0.0527 ± 0.0074	0.0491 ± 0.0064	0.2495							
AP CoM Velocity Range	0.0944 ± 0.0099	0.1002 ± 0.0113	0.0874 ± 0.0101	0.0086*	0.2071	0.1396		0.0808		0.0022*	0.2739
ML CoM Velocity Range	0.0845 ± 0.0116	0.0825 ± 0.0108	0.084 ± 0.0108	0.8732							
VT CoM Velocity Range	0.1692 ± 0.0271	0.1913 ± 0.0275	0.1752 ± 0.025	0.0742							
AP CoM Acceleration Range	0.4186 ± 0.0597	0.4751 ± 0.0662	0.4193 ± 0.0509	0.0185*	0.1769	0.0129*	0.1768	0.9761		0.0155*	0.1917
ML CoM Acceleration Range	0.1639 ± 0.0177	0.185 ± 0.0254	0.1801 ± 0.0148	0.0156*	0.1836	0.0058*	0.2001	0.0344		0.5074	
VT CoM Acceleration Range	0.6275 ± 0.0867	0.713 ± 0.1001	0.6789 ± 0.0838	0.0437*	0.1416	0.0134*	0.1826	0.1345		0.3174	
AP Trunk Angle Range	3.0408 ± 0.7662	2.9569 ± 0.7027	2.7233 ± 0.7378	0.4938							
ML Trunk Angle Range	2.482 ± 0.7381	2.5143 ± 1.0978	3.0228 ± 1.0312	0.2559							
AP Trunk Omega Range	0.0007 ± 0.0002	0.0009 ± 0.0005	0.0006 ± 0.0002	0.0109*	0.1977	0.0336		0.363		0.0037*	0.2072
ML Trunk Omega Range	0.0005 ± 0.0001	0.0006 ± 0.0003	0.0004 ± 0.0002	0.2135							
Mean AP Trunk Angle	4.5042 ± 1.8298	4.7421 ± 2.1532	5.7943 ± 2.5767	0.2584							
Stride Time	3.5145 ± 0.2253	3.4045 ± 0.2352	3.4186 ± 0.2329	0.3766							
Stance Time	2.1198 ± 0.1546	2.0161 ± 0.1665	2.0988 ± 0.1621	0.1899							
Speed	0.5128 ± 0.041	0.5283 ± 0.0346	0.5086 ± 0.0355	0.3279							
Stride Length	1.7711 ± 0.0945	1.7677 ± 0.0787	1.7106 ± 0.0823	0.1166							
Step Width	0.0988 ± 0.0457	0.0832 ± 0.0514	0.0932 ± 0.0533	0.6910							
Stride Time Variability	0.0802 ± 0.0169	0.0809 ± 0.0219	0.0861 ± 0.0308	0.7688							
Stance Time Variability	0.0987 ± 0.0214	0.0907 ± 0.025	0.0693 ± 0.024	0.0051*	0.2269	0.3602		0.0017*	0.3107	0.0183*	0.1703
Speed Variability	0.0214 ± 0.0041	0.0228 ± 0.005	0.0219 ± 0.0071	0.7794							
Stride Length Variability	0.0601 ± 0.0097	0.0673 ± 0.0252	0.0705 ± 0.0254	0.4111							
Step Width Variability	0.0346 ± 0.0037	0.0364 ± 0.0066	0.0373 ± 0.0074	0.4604							
Min Clearance Mean	0.002 ± 0.0008	0.0026 ± 0.0026	0.002 ± 0.0013	0.5889							
Min Clearance Variability	0.0043 ± 0.0049	0.004 ± 0.0067	0.0034 ± 0.0102	0.9470							
Max Clearance Variability	0.0071 ± 0.0073	0.0098 ± 0.0084	0.013 ± 0.009	0.1685							
AP CoP Range	0.1858 ± 0.0158	0.189 ± 0.0124	0.1883 ± 0.0118	0.7035							
ML CoP Range	0.0129 ± 0.0038	0.0135 ± 0.0057	0.013 ± 0.0056	0.9067							
ML CoP Peak	0.0118 ± 0.0035	0.0114 ± 0.0042	0.0106 ± 0.004	0.5951							
ML CoP Mean	0.0068 ± 0.0025	0.0055 ± 0.0025	0.0049 ± 0.0024	0.0442*	0.0972	0.0989		0.0188		0.4743	
Heel Strike Force	1.0552 ± 0.0616	1.0467 ± 0.0737	0.9934 ± 0.0713	0.1177							
Push Off Force	1.0488 ± 0.1167	1.0336 ± 0.1011	1.0325 ± 0.114	0.9241							

Table S2. Summary table of all measures and statistics in the study for condition S (N = 15), condition W1 (N = 15), and condition W2 (N = 14). Values in the condition column represent the subject mean and standard deviation. The P column represent the p-value produced from a one-way ANOVA ($*p < 0.05$). The t-test column contains the p-values produced from the post-hoc Holm-Sidak t-test ($\alpha = 0.05$). The η^2 columns represents the effect size calculated for statistically significant results of the ANOVA or t-test to the left of each η^2 column.

AP Stepping Regression Statistics						
Regression Feature	Summer Value	Winter Value	Summer (p-value)	Winter (p-value)	P	η^2
x	0.9419 ± 0.0968	1.0123 ± 0.0546	1.7845e-15*	2.2317e-19*	0.0207*	0.1706
y	0.0197 ± 0.1254	-0.0093 ± 0.1446	0.5518	0.8067	0.5613	
z	2.3107 ± 0.5734	3.197 ± 0.8955	3.0072e-10*	1.4874e-09*	0.0031*	0.2712
$\dot{x}$	1.0995 ± 0.1833	1.025 ± 0.2389	1.3992e-12*	1.3063e-10*	0.3461	
$\dot{y}$	0.1515 ± 0.1315	0.1711 ± 0.1799	0.0005*	0.0025*	0.7356	
$\dot{z}$	0.4679 ± 0.2383	0.659 ± 0.1935	2.4598e-06*	2.7611e-09*	0.0227*	0.1719
$\ddot{x}$	0.3046 ± 0.1784	0.303 ± 0.1999	1.1661e-05*	4.0657e-05*	0.9812	
$\ddot{y}$	0.1147 ± 0.0933	0.0702 ± 0.1333	0.0003*	0.0608*	0.2985	
$\ddot{z}$	0.1645 ± 0.0974	0.1407 ± 0.0703	1.3168e-05*	1.9570e-06*	0.4499	
R^2	0.9277 ± 0.029	0.9025 ± 0.0648			0.1816	
ML Stepping Regression Statistics						
Regression Feature	Summer Value	Winter Value	Summer (p-value)	Winter (p-value)	P	η^2
x	-0.0317 ± 0.0526	0.0155 ± 0.049	0.0349*	0.2406	0.0167*	0.1878
y	0.5928 ± 0.1384	0.629 ± 0.1175	1.3286e-10*	6.5683e-12*	0.4466	
z	-0.0598 ± 0.4789	0.0579 ± 0.3326	0.6362	0.5108	0.4407	
$\dot{x}$	0.0776 ± 0.1098	0.0215 ± 0.0752	0.0160*	0.2874	0.1136	
$\dot{y}$	1.8308 ± 0.2287	1.8401 ± 0.2043	2.6466e-14*	5.1973e-15*	0.9075	
$\dot{z}$	0.0075 ± 0.1405	0.0063 ± 0.1525	0.8394	0.8742	0.9831	
$\ddot{x}$	0.0818 ± 0.1107	0.0324 ± 0.1056	0.0125*	0.2550	0.2208	
$\ddot{y}$	0.1706 ± 0.1293	0.177 ± 0.1609	0.0002*	0.0008*	0.9062	
$\ddot{z}$	0.0146 ± 0.0588	0.0027 ± 0.055	0.3530	0.8543	0.5708	
R^2	0.6495 ± 0.1089	0.6017 ± 0.0921			0.2042	

Table S3. Summary table of the gains and R^2 values of the stepping regressions for the summer and winter trials. Summer and winter columns represent the subject mean and standard deviation of the gains and R^2. Summer and winter p-value columns represent the p-value produced from a t-test to determine if the gain was statistically different from zero (*$p < 0.05$). The P column represents the p-value produced from the ANOVA comparing summer and winter regression values (*$p < 0.05$). The η^2 column represents the effect size calculated for statistically significant results of the one-way ANOVA.

Regression Feature	Summer Value	Winter Value	Summer (p-value)	Winter (p-value)	P	η^2
Ankle	-0.0353 ± 0.4052	0.028 ± 0.3842	0.7406	0.8322	0.7091	
Trunk	0.781 ± 0.3736	0.9382 ± 0.3993	1.1908e-06*	0.0001*	0.3412	
R^2	0.1463 ± 0.1089	0.1275 ± 0.0796			0.6570	

Table S4. Summary table of the gains and R^2 values of the ankle and trunk recovery regressions for the summer and winter trials. Summer and winter columns represent the subject mean and standard deviation of the gains and R^2. Summer and winter P value columns represent the p-value produced from a t-test to determine if the gain is statistically different from zero (*$p < 0.05$). The P column represents the the p-value produced from the comparison between the values from the summer and winter regression (*$p < 0.05$). The η^2 column represents the effect size calculated for statistically significant results of the one-way ANOVA.
